# Etiopathogenic features of severe epistaxis in histological samples from individuals with or without arterial hypertension

**DOI:** 10.1038/s41598-022-05278-9

**Published:** 2022-01-25

**Authors:** Gustavo Lara Rezende, Leonel Alves Oliveira, Renata Oliveira Soares, Fabiana Pirani Carneiro, Marcio Nakanishi, Sônia Nair Baó, André Luiz Lopes Sampaio, Selma Aparecida Souza Kückelhaus

**Affiliations:** 1 Department of Otolaryngology, Hospital de Base, Brasilia, Federal District, Brazil; 2 Nucleus of Research in Applied Morphology and Immunology, Faculty of Medicine, University of Brasilia, Federal District, Brazil

**Keywords:** Light-sheet microscopy, Scanning electron microscopy, Anatomy, Cardiology

## Abstract

There is a consensus that arterial hypertension (AH) is associated with stroke. Therefore, this study aimed to evaluate the histology of the microvasculature associated with the mucosa of the posterior nasal cavity to identify possible factors related to vascular weakening and rupture. Histological sections were obtained from hypertensive and normotensive individuals, regardless of epistaxis. Our results showed that the group with AH had: (a) smaller median diameter of the lumen of arteries and arterioles; (b) increased thickness of the intimal arteries and arterioles, slight inflammatory infiltrate, and rupture of internal elastic lamina; (c) greater thickness of the middle tunica in arterioles; (d) lower percentage of histological sections with non-injured intimal layers in capillaries, arterioles, and small arteries; (e) lower percentage of histological sections with intact media tunic and/or myocytes juxtaposed in arteries and arterioles; (f) no difference between the diameters of small arteries or arterioles. The intima was thicker in individuals with severe epistaxis than in the normotensive group, but it did not differ from the AH group. Thus, hypertension may cause structural lesions in the vascular layers, and in the absence of tissue repair and the persistence of AH, these lesions may favour vascular rupture, especially during hypertensive peaks.

## Introduction

Epistaxis is an example of an otorhinolaryngological emergency which affects 6% of the population^1^. It is mainly caused by the rupture of the sphenopalatine artery or its branches, and treatment requires immediate surgical intervention in 5% of cases^2^. It is believed that the origin of severe epistaxis is idiopathic in more than 80% of cases^3^, but some studies have suggested that it is associated with diabetes, progressive vascular obstruction, and systemic arterial hypertension (AH)^4–7^. However, the aetiology still remains unknown.

The vascular network of the nasal mucosa consists of small-calibre veins derived from the sphenopalatine artery emerging from the sphenopalatine foramen and branching into a vascular bed which irrigates the entire posterior lateral nasal wall^8^. Considering that small arteries and arterioles help to reduce pre-capillary pressure for metabolic changes, structural alterations of the vessels may compromise their integrity and favour rupture.

In AH, structural changes are initially observed in the microvasculature and in vessels of greater calibre to adapt to the increased blood pressure, however, prolonged alterations may lead to vessel weakening and rupture^9–12^. These changes can result in the formation of the atheroma plaque (lipid deposit) in the tunica intima of medium- and large-calibre arteries, as well as arteriolosclerosis, caused by the thickening and hardening of the vascular wall of small arteries and arterioles. In both pathological processes, vascular weakening may evolve into the rupture of the compromised vessel, as observed in organs such as the heart, brain, kidneys, and limb extremities of individuals with AH^5,13^.

The association of hypertension with severe epistaxis and its role in the structure of small arteries, arterioles and capillaries remains unclear. Therefore, this study sought to evaluate the structure of the posterior nasal cavity microvasculature of individuals with or without any history of systemic arterial hypertension to identify possible factors related to vascular weakening and rupture. Furthermore, we analysed the structural changes in the posterior nasal mucosal vessels of patients who underwent surgery for severe epistaxis in an attempt to determine the related factors.

## Results

There was no age difference between the hypertensive group (63 ± 13) when compared to the normotensive group (53 ± 11) (t-test; *p* > 0.05) (Table 1).

**Table 1:** Epidemiological profile of normotensive and hypertensive individuals, containing cause of death.

Normotensive			Hypertensive			
Individual	Age (Years)	Causa mortis	Individual	Age (Years)	Medication for SAH*	Causa mortis
1F	50	Hemorrhagic stroke	1F	55	P	Chronic hepatitis
2F	58	Chagasic cardiomyopathy	2F	60	P	Hemorrhagic stroke
3F	56	Cervical tumor	3F	53	C	Cerebral tumor
4F	**45**	–	4F	58	C + M	Congestive heart failure
5M	55	Chagasic cardiomyopathy	5F	62	C	Mesenteric thrombosis
6M	42	Pneumonia	6F	64	C	Ischemic stroke
7M	40	Prostate tumor	7F	**45**	**C**	–
8M	69	Cerebral abscess	8M	59	P	Hemorrhagic stroke
9M	74	Pneumonia	9M	78	P	Thyroid tumor
10M	**43**	–	10M	52	C	Hemorrhagic stroke
11M	**45**	–	11M	87	C + H	Bone tumor
			12M	**81**	**C**	–
			13M	**55**	**C**	–
Mean ± SD	53 ± 11		Mean ± SD	64 ± 13		

*M* = Male, *F* = Female; Bold pairs = Different values (p < 0.05); (*) Captopril (C), Propranolol (P), Hidrochlorothiazide (H), Metropolol (M).

Among normotensive individuals, death occurred due to infectious processes (62.5%), tumours (25%), and haemorrhage (12.5%), while in the hypertensive group, death occurred predominantly due to cardiovascular diseases (60%), but also due to tumours (30%) and infection (10%). There was no significant difference in the proportions between the two groups in relation to death from vascular causes (Fisher, *p* > 0.05) (Table 1).

**Figure 1:**
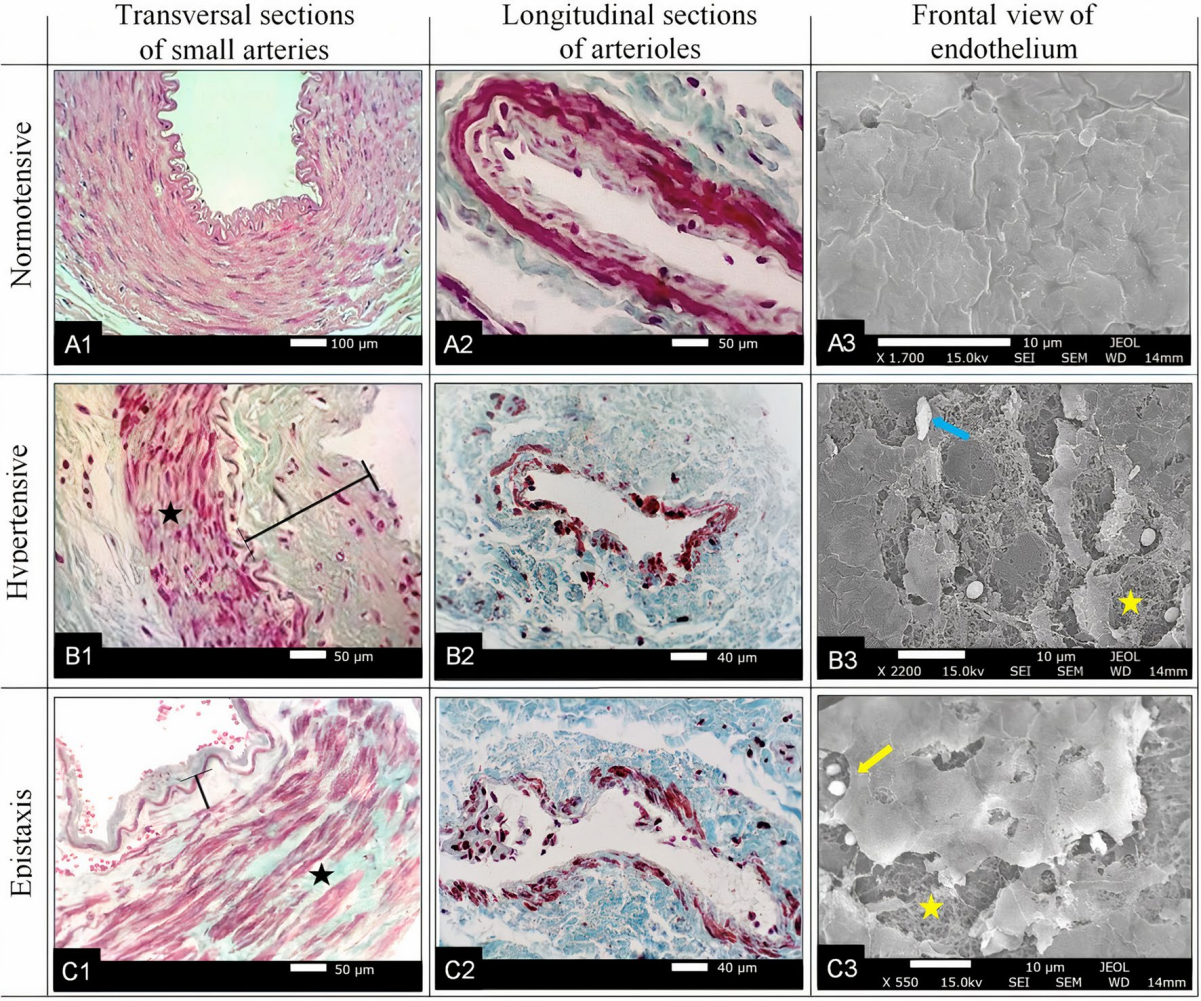
Photomicrographs and scanning electromicrographs obtained from fragments of the posterior nasal mucosa of normotensive individuals, hypertensive individuals and which are representative of the structural evaluation of the branches of the sphenopalatine artery. Observe the normal layers (**A1**, Hematoxilin & Eosin; **A2**, Gomori) and arteries exhibit areas in which muscle cells lost juxtaposition and greater presence of extracellular matrix (black star) (**B1**,**B2**,**C1**,**C2**; Gomori Trichrome) and larger intimal layer (black bars) (**B1**,**C1**). The third column shows the frontal view of the intima from three individuals (**A3** = normotensive; **B3** = hypertensive; **C3** = epistaxis) showing that the endothelial cells are adhered and continuous in normotensive (**A3**), whereas in (**B3**,**C3**) it is possible to observe regions with endothelial loss (yellow star), platelets (yellow arrow) and a leucocyte (blue arrow) attached to the lamina propria.

To determine the differences between the hypertensive and normotensive groups, the diameters of arterioles and small-calibre arteries, their lumens, the thickness, and the integrity of the vascular tunics (intima and medium) were evaluated. In comparison with normotensive individuals, hypertensive individuals presented: (a) smaller median diameter of the lumen of arteries (39 vs. 188 µm) and arterioles (40 vs. 49 µm) (Unpaired t test; *p* = 0.014); (b) increased thickness of the intimal arteries (31 vs. 8 µm) (Unpaired t test; *p* = 0.010) and arterioles (11 vs. 6 µm) (Unpaired t test; *p* = 0.009) (Fig. 1A1,B1); (c) greater thickness of the middle tunica in arterioles (72 vs. 49 µm) (Unpaired t test; *p* = 0.017) (Fig. 1A1,B1, 2C1); (d) a lower percentage of histological sections with non-injured intimal layers in capillaries (Mann–Whitney; *p* = 0.003), arterioles (Mann–Whitney; *p* = 0.001) and small arteries (Unpaired t test; *p* = 0.001) (Figs. 1A1-3,B1-3, 2B); (e) lower percentage of histological sections with intact media tunic and/or myocytes juxtaposed in arteries (Mann–Whitney; *p* < 0.001) and arterioles (Mann–Whitney; *p* = 0.004) (Figs. 1A1-3,B1-3, 2D); (f) no difference between the diameters of small arteries or arterioles (Unpaired t test; *p* > 0.05) (Table 2). Semi-quantitative analyses revealed slight inflammatory infiltrate of macrophages and lymphocytes in hypertensive individuals, as well as rupture of the internal elastic lamina (Fig. 1A1-3,B1-3).

**Figure 2:**
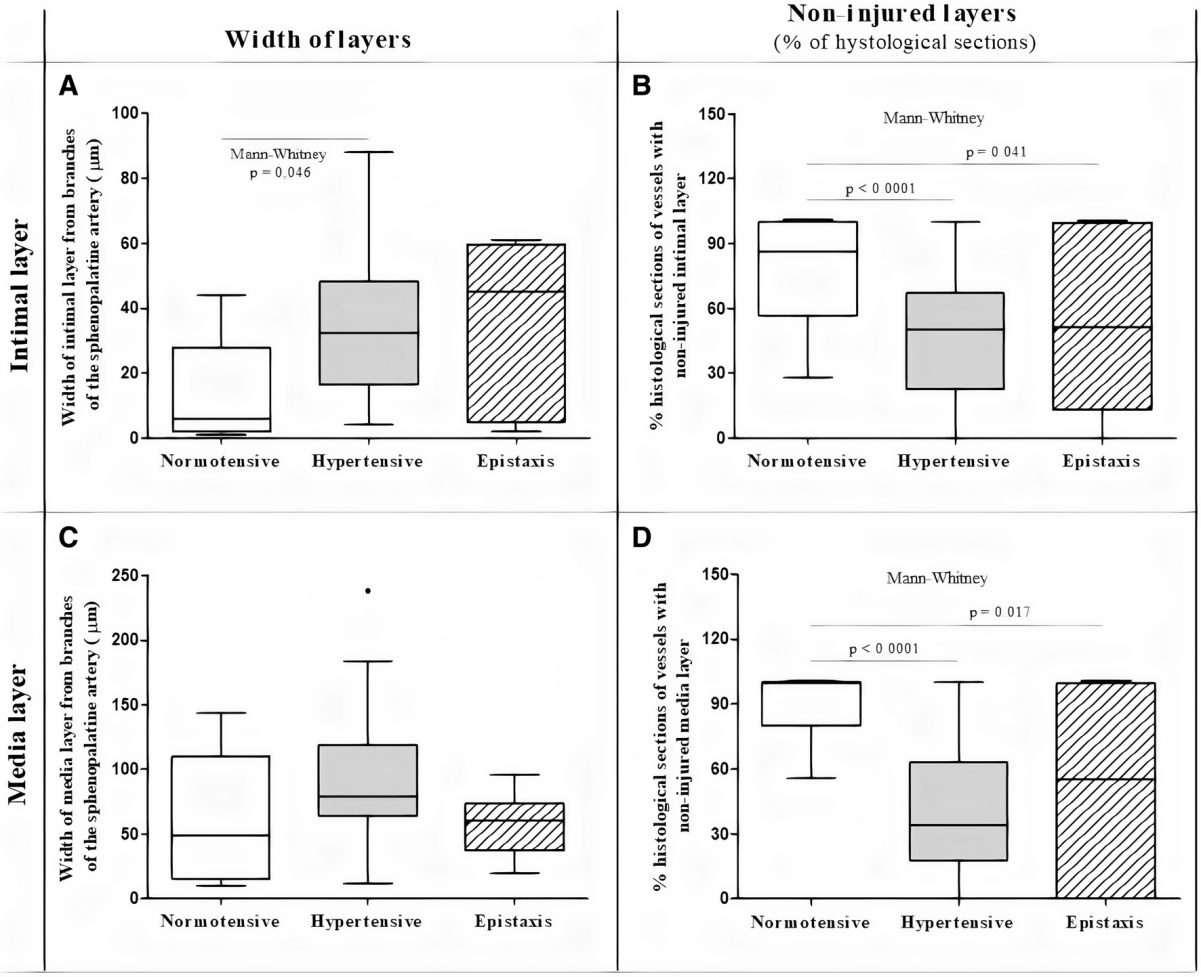
Width of intima (**A**) or media layer (**B**) and percentage of non-injured intima (**C**) or media layer (**D**) of sphenopalatine branches from normotensive, hypertensive and epistaxis groups. Results analyzed by t-test or Mann–Whitney showed that the width of intima to normotensive was lower than hypertensive (*p* = 0.046), but not to the epistaxis (*p* > 0.05); the width of media did not differ between three groups (*p* > 0.05). The normotensive presented higher % of non-injured intima (**C**) or media (**D**) when compared to hypertensive or epistaxis group (*p* < 0,05); hypertensive and epistaxis group did not differ in all analyzes (Unpaired t test, *p* > 0.05). Results are represented as medians, quartiles, maximum and minimum and extreme values (Prism 5.0, GraphPad, Software, Inc., San Diego, CA, USA: https://www.graphpad.com/scientific-software/prism).

**Table 2:** Morphometric data obtained from the microvasculature of the posterior nasal cavity of individuals with or without systemic arterial hypertension.

Parameters	SAH	Median (minimum to maximum)		
		Capilary	Arteriola	Artery
**Diameter**				
Vessel	No	12 (12 to 14)	163 (120 to 223)	890 (617 to 1227)
	Yes	13 (12 to 14)	195 (165 to 218)	951 (414 to 1109)
Lumen	No	9 (7 to 10)	**49** (35 to 89)	**188** (120 to 351)
	Yes	8 (7 to 10)	**40** (33 to 54)	**39** (34 to 47)
**Width of layer**				
Intima	No	5 (4 to 5)	**6** (5 to 7)	**8** (7 to 12)
	Yes	5 (4 to 6)	**11** (9 to 17)	**31** (14 to 50)
Media	No	–	**49** (33 to 79)	**309** (248 to 485)
	Yes	–	**72** (66 to 85)	**457** (190 to 535)
**% non-injured histological sections**				
Intima	No	**99** (93 to 100)	**86** (52 to 100)	**70** (51 to 90)
	Yes	**66** (39 to 73)	**50** (9 to 59)	**34** (0.3 to 55)
Media	No	–	99 (86 to 100)	99 (71 to 100)
	Yes	–	66 (46 to 77)	32 (17 to 36)

Bold pairs = Different values (*p* < 0.05).

When we evaluated the specimens from the six patients who underwent surgery for severe epistaxis and compared them to the normotensive (n = 8) and hypertensive (n = 10) groups, we observed that the median width of the intima was greater in the hypertensive group (25.0 µm) than in the normotensive group (6.5 µm), but it did not differ from the epistaxis patients (45.0 µm) (unpaired t test, *p* > 0.05) (Fig. 2A); the median width of the media layer did not differ among the three groups (Kruskal–Wallis; *p* > 0.05) (Fig. 2C).

The results also showed that the normotensive group had a higher percentage of non-injured histological sections of the intima (97%) or media (98%) layer than the hypertensive group (intima = 42%; media = 43%) or the epistaxis group (intima = 52%; media = 56%) (Mann–Whitney, *p* < 0.05), and there was no difference between hypertensive and epistaxis groups (unpaired t-tests, *p* > 0.05) (Figs. 1A1-3,B1-3,C1-3, 2).

## Discussion

The present study evaluated the morphological structure of capillaries, arterioles, and small-calibre arteries, and branches of the sphenopalatine artery to identify possible factors related to weakening and vascular rupture in this region.

This study may be the first histological model to describe the importance of thickening of intima-media arterial layers and disruption of juxtaposition myocytes on the posterior nasal microvasculature between hypertensive and normotensive individuals. These histological injuries of the AH are described in the medical literature as glomerulosclerosis^14^, stroke^15^, and peripheral vascular disease^16^, but unclear as epistaxis. Based on our findings, we believe that hypertension might be both a cause and a consequence of possible central artery stiffening, which in turn is an initiator and indicator of possible vascular rupture. Stiffening can occur due to remodelling of the nasal arterial wall, which is driven by mechanical stimuli and mediated by inflammatory signals of hypertension, leading to changes in extracellular matrix composition and organisation, promoting weakening of artery walls.

The concomitant presence of a long history of hypertension may generate intimal thickening and luminal narrowing of arteries and arterioles, causing damage to many end organs^17^. The severely fragmented internal elastic lamina and thin or absent tunica media are associated with cerebral aneurysms^18^. However, our samples did not demonstrate any dilations on these weak points along the arterial circulation. Moreover, the fragmented internal elastic lamina acts as the starting point for vascular smooth muscle cells migrating to the blood vessels and thickening of the tunica media involved in vascular remodelling during the development of systemic AH as we observed^15^.

According to the criteria of the American Heart Association^19^, recent lesions characterised by intima thickening and mild inflammatory infiltrate (atheromatosis, grade < III), such as those observed in our hypertensive group, are frequent^19,20^ and generally do not present the risk of rupture, but may reflect the normal remodelling observed in aging^19,21^ and even result in changes in blood flow in these patients. However, the reduction of elastic fibres with subsequent collagen accumulation leads to arterial stiffening and intima-media thickening, which are independent predictors of incident hypertension in prospective community-based studies^22^. Kunz et al.^23^ mentioned that it is crucial to identify individuals with an increased atherosclerotic burden before such life-threatening events take place. The occurrence of severe epistaxis in his study was shown to be closely associated with the prevalence of atherosclerotic cardiovascular disease by analysing the carotid intima-media thickness by means of an ultrasonographic study^23^.

A possible effect of hypertension on the exacerbation of intrinsic vascular injury processes was demonstrated by the low percentages of integrity/juxtaposition of the intima in hypertensive individuals (15%), compared to those in normotensive individuals (83%) in our study. These findings are similar to those of studies on carotid arteries, demonstrating that shearing on the endothelium by deviating vascular flow decreases the juxtaposition of smooth muscle cells by increasing vascular wall fragility^24^.

The morphometric analyses showed that the group of individuals who underwent surgery for severe epistaxis presented the width of the intima and percentage of histological sections with non-injured intima and media similar to those observed for the group of hypertensive patients. Although the direct connection of epistaxis and cardiovascular diseases has not yet been made, these changes could lead to friability of the nasal vessels and therefore a predisposition for nosebleeds^23^. This might be especially important in the posterior nasal mucosa, because during its course, the sphenopalatine artery and/or its branches are directly related to the ethmoidal crest of the palate and the Choana Arch, where the main changes in the direction of the vascular path are observed^25^. The frequent rupture of the vessels in these regions reported in some studies may be favoured by vascular fragility from different causes, especially during hypertensive peaks^2,8,26^.

The presence of cerebral microbleeds is considered a strong predictor of future stroke, particularly haemorrhagic stroke. Ding et al.^27^ performed a systematic review and meta-analysis and found that there is a relationship between large artery atherosclerosis and cerebral microbleeds. Isolated medial layer thickness reflects not only early atherosclerosis but also nonatherosclerotic compensatory remodelling with large medial hypertrophy as a result of smooth muscle cell hyperplasia and fibrocellular hypertrophy^28^. This demonstrates that our findings of no statistical difference between isolated media layer measurement in the hypertensive group and normotensive group of patients who suffered epistaxis, might be due to hypertension atherosclerosis, as seen in early plaque formation from thickening of the intima-media in AH.

In spite of the convenience sample, mostly the cadavers, it was not possible to categorise the hypertensive group according to the duration of hypertension, or as still adhered to, while they were alive. However, it is understood that individuals were eligible for the study because they were followed up and treated for systemic arterial hypertension at the Health Department of the Federal District in accordance with international protocols at the time (Eighth Joint National Committee)^29^. Another important limitation of the present study was the removal of the sphenopalatine artery. Portable videoendoscopy equipment was used, and complex dissection was performed by experienced surgeons to locate and remove arterial branches without histological damage. In this way, these samples are difficult to obtain even in large study centres.

We showed that hypertension causes structural lesions in the intima and media layers, which weaken the vessels of the nasal mucosa. These structural lesions on the nasal vessels could provide a histological model for further studies on epistaxis.

Severe epistaxis has been shown to be closely associated with the prevalence of atherosclerotic cardiovascular disease caused by AH^29^. Accordingly, patients affected by epistaxis should be regarded as having an elevated cardiovascular risk, which indicates the need for appropriate further medical assessment and preventive measures. This confirms that severe epistaxis can be part of the AH diagnosis, and otolaryngologists should be involved in the diagnosis and initiation of anti-hypertensive and anti-aggregant drug treatment when necessary.

The controversy regarding hypertension as a possible etiopathogenesis needs to be elucidated by scientific research.

## Methods

### Individuals

This study was performed with convenient samples collected from 24 individuals, with 18 obtained from fresh cadavers and six from individuals who suffered severe epistaxis and required surgical treatment (Table 1).

Data were collected from the medical records of patients or family members of the cadavers, and the individuals were divided into three groups (Table 2): (1) 8 cadavers without AH and no other chronic disease reported, named as *normotensive*; (2) 10 cadavers with AH and under medical treatment, named *hypertensive*; (3) 6 live patients affected by severe epistaxis who underwent thermoelectric cauterisation of the injured vascular segment, named as *epistaxis* (three presented with SAH); none of these patients reported chronic disease or any comorbidity.

The present study was performed strictly adhering to the ethical standards for scientific research with humans under Law 6638/79 and according to the Declaration of Helsinki. Fresh cadavers were obtained from the Institute of Forensic Medicine of the Federal District, Brazil, in accordance with the protocol for the disposal of human body parts for teaching and research. Individuals with facial trauma, coagulopathies, allergies, diabetes mellitus, and dyslipidaemia were excluded.

The study was approved by the Research Ethics Committee of the School of Health of the Federal District, Brasília, Brazil (Protocol No. 152/2012). Signed informed consent was obtained from all patients and those responsible for the cadavers included in this study.

### Obtaining samples of the nasal mucosa and segments of the sphenopalatine artery

For histological description and comparison, samples were obtained by dissection and removal of the mucosa of the posterior nasal cavity and segments of the sphenopalatine artery and its branches, with the exit of the artery from the sphenopalatine foramen. An approximate extension of 1 cm^2^ and a complete depth of the nasal mucosa flap were removed. The procedures were recorded by nasal video endoscopy with zero grade 4.0 mm optics (Karl Storz, Tuttlingen, Germany). Samples from cadavers were collected up to 3 h after death at the Anatomical Service of the Hospital de Base, Brasília, Federal District.

### Obtaining histological images

After the samples were obtained as specified above, the histological specimens were fixed in 4% paraformaldehyde, dehydrated in alcohol (70%, 80%, 90%, and 3 × 100%), diaphanized in xylol (2 × for 30 min), impregnated, and placed in paraffin at 60 °C, at the Histological Laboratory of Medicine Faculty, University of Brasília. Half of the segments were stained with haematoxylin & eosin and the other half with the Gomori technique. The specimens were frontally positioned in paraffin blocks, integrally sectioned (5 μm-thick slices), and the histological sections were stained with haematoxylin and eosin or Gomori's trichrome. Subsequently, the images were obtained using the Aperio photodocumentation system (Leica, Newcastle, England) which allows for full scanning of histological sections on a slide, with a capture resolution of up to 40× magnification. Histological analyses were performed using the scanned sections in the ImageScope software (version 12,105,029) (Leica, Newcastle, England) which allows for the standardisation of the analyses. For this, magnification (up to 1000×), area selection, and measurements were used. To identify the basal membranes of endothelial cells or myocytes, 25% of the histological sections of each individual were stained with the reticulin technique.

For analysis using scanning electron microscopy (JEOL 7001F, Tokyo, Japan), fragments of the arterial branches of the posterior nasal mucosa were fixed in 2% glutaraldehyde solution, 2% paraformaldehyde, and in 0.1 M sodium cacodylate buffer with a pH of 7.2. After fixation, they were washed in 0.1 M sodium cacodylate buffer and post-fixed in 1% osmium tetroxide and sodium cacodylate buffer. The specimens were then dehydrated for 15 min in solutions with increasing concentrations of acetone (30%, 50%, 70%, 90%, and 3 × 100%). Then, the specimens were dried to the critical point with CO_2_ and metallised with gold.

#### Evaluation of layer thickness and vascular diameter

To measure the thickness of the vascular layers, the total diameter and lumen of the vessels, cross-sections of small-calibre arteries (> 200 to 1500 μm in diameter, consisting of intima, media, and adventitia to 47.8 ± 12.9 μm in length), arterioles (> 16 to 200 μm, consisting of intima, media, and adventitia), and capillaries (10 to 15 μm, consisting of the endothelium and basal membrane) in an extension equivalent to 60.0 ± 12.5 μm were selected. The measurements were taken at four equidistant points to determine the mean value of the histological sections.

#### Evaluation of vascular segment integrity

Qualitative analyses of the vascular integrity were performed in the cross-sections of capillaries, arterioles, and small-calibre arteries. In the tunica intima, the juxtaposition of endothelial cells and continuity of the basal membrane were evaluated. The adhesion between smooth muscle cells was evaluated for the middle tunica (arterioles and arteries). For comparisons between the study groups, the numbers 0 (zero) or 1 (one) were assigned when the trait analysed in each histological section was absent or present, respectively. The results were expressed as percentages (%) of histological sections which presented the characteristics analysed, and were tabulated and grouped and later analysed in the statistical program for comparisons between the normotensive and hypertensive groups.

### Connective tissue analyses

For the semi-quantitative analysis, inflammatory cells (neutrophils, eosinophils, macrophages, or lymphocytes) were quantified in the mucosa in four equidistant fields equivalent to 400 μm^2^ and after tabulating, the data were compared, and all histological sections were analysed by a single blinded researcher. The measurements were taken on each scanning microphotograph using the Aperio photo documentation system (Leica, Newcastle, England).

### Statistical analysis

The results were evaluated using Bartlett’s test for equal variances and the Kolmogorov–Smirnov test for normal distribution before comparative analysis. The analyses were performed using the ANOVA test, followed by the Student–Newman–Keuls method or Kruskal–Wallis test, and the Dunn’s method to compare multiple unrelated samples of normally or abnormally distributed data. The t-test or Mann–Whitney test was used to compare two unrelated groups with normally or abnormally distributed data, and Fisher's test was used to compare proportions in a contingency table. Statistical tests and graphical presentation of the data were performed using the Prism 5.0 (GraphPad, Software, Inc., San Diego, CA, USA: https://www.graphpad.com/scientific-software/prism), and differences with a two-tailed *p*-value < 0.05, were considered statistically significant.
